# Crustacean zooplankton release copious amounts of dissolved organic matter as taurine in the ocean

**DOI:** 10.1002/lno.10603

**Published:** 2017-06-20

**Authors:** Elisabeth L. Clifford, Dennis A. Hansell, Marta M. Varela, Mar Nieto‐Cid, Gerhard J. Herndl, Eva Sintes

**Affiliations:** ^1^ Department of Limnology and Bio‐Oceanography Center of Ecology, University of Vienna Vienna Austria; ^2^ Department of Ocean Sciences RSMAS University of Miami Miami Florida; ^3^ Centro Oceanográfico de A Coruña IEO, Instituto Español de Oceanografía A Coruña Spain; ^4^ CSIC, Instituto de Investigaciones Marinas de Vigo Vigo Spain; ^5^ NIOZ, Royal Netherlands Institute for Sea Research, Department of Marine Microbiology and Biogeochemistry, Utrecht University Den Burg The Netherlands

## Abstract

Taurine (Tau), an amino acid‐like compound, is present in almost all marine metazoans including crustacean zooplankton. It plays an important physiological role in these organisms and is released into the ambient water throughout their life cycle. However, limited information is available on the release rates by marine organisms, the concentrations and turnover of Tau in the ocean. We determined dissolved free Tau concentrations throughout the water column and its release by abundant crustacean mesozooplankton at two open ocean sites (Gulf of Alaska and North Atlantic). At both locations, the concentrations of dissolved free Tau were in the low nM range (up to 15.7 nM) in epipelagic waters, declining sharply in the mesopelagic to about 0.2 nM and remaining fairly stable throughout the bathypelagic waters. Pacific amphipod–copepod assemblages exhibited lower dissolved free Tau release rates per unit biomass (0.8 ± 0.4 μmol g^−1^ C‐biomass h^−1^) than Atlantic copepods (ranging between 1.3 ± 0.4 μmol g^−1^ C‐biomass h^−1^ and 9.5 ± 2.1 μmol g^−1^ C‐biomass h^−1^), in agreement with the well‐documented inverse relationship between biomass‐normalized excretion rates and body size. Our results indicate that crustacean zooplankton might contribute significantly to the dissolved organic matter flux in marine ecosystems via dissolved free Tau release. Based on the release rates and assuming steady state dissolved free Tau concentrations, turnover times of dissolved free Tau range from 0.05 d to 2.3 d in the upper water column and are therefore similar to those of dissolved free amino acids. This rapid turnover indicates that dissolved free Tau is efficiently consumed in oceanic waters, most likely by heterotrophic bacteria.

Marine dissolved organic matter (DOM) originates primarily from phytoplankton either via extracellular release or via zooplankton grazing and viral lysis (Carlson and Hansell 2014). However, essentially all marine organisms release DOM into the ambient water (Steinberg et al. 2004; Carlson and Hansell 2014). The importance of DOM as a substrate for heterotrophic bacteria is well established (e.g., Carlson and Hansell 2014; Moran et al. 2016). Among other DOM compounds, dissolved free amino acids (DFAA) are released into the ambient water and serve as a major nutrient and energy source for heterotrophic bacterioplankton (Keil and Kirchman 1999; Zubkov et al. 2008; Sarmento et al. 2013). Consequently, DFAA are turned over in euphotic waters in the range of minutes to days (Fuhrman and Ferguson 1986; Simon and Rosenstock 2007; Simon et al. 2012). As a consequence of this rapid turnover, the concentration of DFAA in coastal and open ocean waters is usually in the low nanomolar range in surface waters and decreases with depth, reaching concentrations close to or below the detection limit in the bathypelagic layers (Sipler and Bronk 2015).

In contrast to DFAA in the ocean, taurine (2‐aminoethanesulfonic acid, Tau) is far less studied. Tau is a naturally occurring free non‐protein organic acid with a sulfonic group instead of the carboxyl group characteristic for amino acids. Only a few studies have reported dissolved free Tau concentrations in coastal and near‐shore surface waters, which are typically in the low nanomolar range (up to ∼35 nM) (Mopper and Lindroth 1982; Kuznetsova and Lee 2002; Lu et al. 2014). However, to the best of our knowledge, dissolved free Tau concentrations have not been reported for open oceanic and deep waters.

Tau is synthesized by a wide range of marine organisms, such as fish (Chang et al. 2013), invertebrates (Allen and Garrett 1971; Welborn and Manahan 1995) including crustaceans (Finney 1978), and algae (Amin et al. 2015; Tevatia et al. 2015). The role of Tau as an osmolyte in marine metazoans (Awapara et al. 1959; Awapara 1962; Allen and Garrett 1971) and in prokaryotes (McLaggan and Epstein 1991; Graham and Wilkinson 1992) is well known. Tau plays an essential role in physiological processes such as osmoregulation, cytoprotection, and neuromodulation (Kaya and Sano 1991; Huxtable 1992; Yancey 2005; Carreto and Carignan 2011; Ripps and Shen 2012). Moreover, free and combined Tau might play a role in detoxification (Rosenberg et al. 2006; Koito et al. 2010) and signaling processes (Wang and Douglas, 1997; Amin et al. 2015).

Metazoans can either synthesize Tau from the sulfur‐containing amino acids cysteine or methionine or take it up as a food source. Several studies have reported the presence of Tau in the tissues, as well as in the release products of zooplankton, especially in crustaceans such as copepods and shrimps (Webb and Johannes 1967; Jeffries 1969; van der Meeren et al. 2008) and in their eggs (Wang et al. 2005). Subsequently, Tau can be released into the surrounding waters together with other organic compounds by excretion, during sloppy feeding, or from the fecal pellets and carcasses of metazoans.

In contrast to eukaryotic organisms, bacteria are able to metabolize Tau as a carbon, nitrogen, sulfur, and/or energy source (Cook and Denger 2006). Recent meta‐proteomics and ‐genomics studies have revealed that genes encoding transport proteins responsible for Tau uptake as well as enzymes involved in Tau degradation pathways are widespread in marine bacterioplankton communities in surface (Smith et al. 2013; Williams and Cavicchioli 2014; Luo et al. 2015; Wang et al. 2016) and deep waters (Eloe et al. 2011; Hawley et al. 2014; León‐Zayas et al. 2015). Moreover, members of ubiquitous marine prokaryotic lineages, such as SAR11 and *Rhodobacteraceae*, utilize Tau for growth (Schwalbach et al. 2010; Steindler et al. 2011; Lenk et al. 2012). However, the potential role of Tau as substrate and/or energy source for heterotrophic bacteria in the ocean has not been explored.

The goal of this study was to determine the concentrations of dissolved free Tau from surface to bathypelagic waters at two contrasting open ocean sites, the Gulf of Alaska (GoA) and the North Atlantic (NA). Moreover, we determined the release rates of dissolved free Tau by abundant marine mesozooplankton common in these two oceanic regions and corresponding turnover times.

## Material and methods

### Study area and sampling

**Figure 1:**
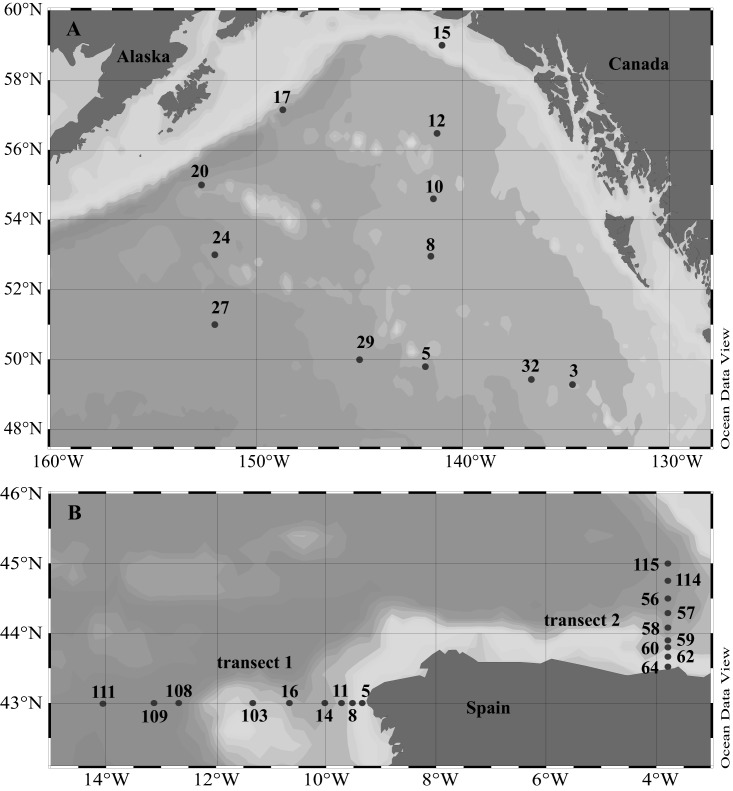
Location of the sampling stations in (**A**) the Gulf of Alaska (GoA) and (**B**) the North Atlantic (NA).

Sampling was conducted in the GoA during the DORC (Deep Ocean Refractory Carbon) research cruise aboard the R/V *Melville* in August 2013 (Fig. 1A) and in the NA during the MODUPLAN cruise aboard the R/V *Sarmiento de Gamboa* in August 2014 (Fig. 1B; transects 1 and 2). Water samples were collected with a CTD (conductivity, temperature, depth) rosette holding 12 L Niskin bottles at 12 and 18 stations on the GoA and NA research cruises, respectively. Seawater was sampled from surface to bathypelagic layers at 6–10 depths per station. Water was collected from the Niskin bottles into 100 mL polycarbonate flasks (acid‐rinsed and three times rinsed with sample water prior to collecting the sample) and immediately processed as described below.

### Determining dissolved free Tau and DFAA concentrations

For determining dissolved free Tau and DFAA concentrations, water was collected from the 100 mL polycarbonate flasks with 20 mL polypropylene syringes (Braun) and filtered through pre‐rinsed 0.2 μm Acrodisc filters (25 mm diameter; Pall, Supor membrane) into pre‐combusted glass vials. Triplicate samples of 5 mL were taken from each depth and stored frozen at −20°C until further analysis using high‐pressure liquid chromatography (HPLC) and fluorescence detection after pre‐column *ortho*‐phthaldialdehyde (OPA) derivatization according to the protocol of Jones et al. (1981), with slight modifications of the mobile phases and gradient as described below.

For the analysis, an Agilent 1260 Infinity Bioinert HPLC System was used, consisting of an autosampler, a quaternary pump, a column oven, and a fluorescence detector. A primary amino acid standard mix was obtained from Agilent Technology. Tau, alpha‐aminobutyric acid (AABA) and gamma‐aminobutyric acid, aspargine, glutamine, tryptophan, methanol, tetrahydrofuran, and sodium acetate trihydrate were obtained from Sigma Aldrich (Germany). The OPA reagent and the borate buffer (0.4 N, pH = 10.2) were purchased from Agilent Technologies (USA).

For open ocean water samples, 1 nM AABA was added as internal standard for the quantification of Tau. Samples with Tau concentrations between the limit of detection (LOD) and limit of quantification (LOQ) were spiked with 1 nM Tau to confirm the identification and quantification. Internal standards should be in the range of the samples, therefore samples from the incubation experiments (described below) were initially measured without the addition of an internal standard to estimate Tau concentrations using external standards. Subsequently, samples were grouped according to their preliminary Tau concentrations and re‐measured with the appropriate amount of internal standard (ranging between 1 nM and 1 μM). Additionally, external standard series were measured for each HPLC run. The linearity of the standard curves was determined by least‐squares linear regression analysis. Derivatization was carried out with the robotic autosampler. Five μL OPA reagent and 75 μL borate buffer were added to 1 mL sample (consisting of 990 μL of sample and 10 μL internal standard) and subsequently mixed. After 2 min of reaction time at room temperature, 500 μL of the reaction mixture were injected into the HPLC. The fluorescent derivatives were separated on a Zorbax ECLIPSE AAA column (4.6 × 150 mm, 3.5 μm) with a Zorbax ECLIPSE AAA guard cartridge (4.6 × 150 mm, 5 μm), the column temperature set at 25°C and a flow rate of 0.8 mL min^−1^. Excitation and emission wavelengths were 340 nm and 450 nm, respectively. An example of the separation of amino acids using the standard mix including Tau (1 μM, gain 10) is shown in Supporting Information Fig. S1. Mobile phases were 50 mM sodium acetate (pH = 6.8) and methanol. Tetrahydrofuran was added to facilitate the separation of the individual compounds. The elution gradient program used in this study for detecting and quantifying amino acids and Tau is shown in Supporting Information Table S1.

The accuracy and precision of the method to determine Tau concentrations were assessed as detailed below. Individual test samples were amended with six concentrations of Tau (1 nM, 5 nM, 10 nM, 500 nM, 1000 nM, and 1500 nM). Five replicates for each concentration were measured. The recovery R (in %) was estimated as R=(Ca−Cu)/Cs×100, where Ca and Cu are the concentrations of Tau in amended and unamended samples, respectively, and Cs is the concentration of the Tau standard added to the test samples. The precision of the method was determined by calculating the relative standard deviation (RSD %) for the replicates. To test the accuracy and precision of the spiked low‐concentration samples, eight replicates of test samples of known concentration (0.25 nM Tau) as well as spiked test samples (spiked with 1 nM Tau, i.e., 0.25 + 1 nM Tau) were measured. Replicates were randomly positioned in the autoanalyzer covering an entire run of measurements (beginning, middle, and end). LOD and LOQ were determined by measuring sequential dilutions of Tau standards until the signal‐to‐noise ratios ≥3 and ≥10 were reached, respectively. *R* ranged from 99% to 111% with an accuracy of 0.25 ± 0.001 (not spiked) and 1.25 ± 0.003 (spiked). The correlation coefficient (*R* ^2^) ranged between 99.7% and 99.9%, the LOD and LOQ varied between 0.01 to 0.02 (nM) and 0.1 to 0.2 (nM), respectively. For more details see Supporting Information Table S2.

### Determining dissolved organic carbon (DOC) and nitrogen (DON)

Dissolved organic carbon (DOC) and total dissolved nitrogen (TDN) concentrations from water samples were analyzed simultaneously using a Shimadzu TOC‐V_CSH_ analyzer coupled with a TNM‐1 Total Nitrogen Analyzer as previously described (Dickson et al. 2007). Nitrite and nitrate concentrations were determined according to Braman and Hendrix (1989) and ammonium as described in Holmes et al. (1999) on the samples collected at the GoA. DON concentrations from GoA samples were calculated as the difference between the TDN and the sum of the dissolved inorganic N‐species (NO_3_‐N, NO_2_‐N, and NH_4_‐N).

### Determining dissolved free Tau and DFAA release by crustacean mesozooplankton

Copepod‐amphipod assemblages were incubated during the GoA cruise at six stations (Sta. 5, 8, 12, 17, 20, 29, see Fig. 1A). During the NA cruise, incubation experiments were conducted with mixed copepod communities at Sta. 14 (see Fig. 1B), and with single copepod species (*Acartia* sp., *Centropagus* sp., *Calanus* sp., *Clausocalanus* sp., *Paracalanus/Pseudocalanus* sp.) at three stations (Sta. 59, 111, and 115, see Fig. 1B). Dissolved free Tau and DFAA concentrations as well as bacterial abundance were monitored in the incubations over 24 h and 8 h in the GoA cruise and NA cruise, respectively. As dissolved free Tau and/or DFAA concentrations declined or remained stable after 8–10 h in the incubations conducted during the GoA cruise, in the NA cruise the incubation time and the sampling intervals were kept shorter.

Zooplankton samples were collected using integrated vertical plankton tows. One or two tows per station were performed depending on the amount of zooplankton specimens collected (Tables 1, 2). The zooplankton net (200 μm mesh size) was hoisted at 30 m min^−1^ in the GoA and at 15 m min^−1^ in the NA from ∼600 m and ∼200 m depth to the surface in the GoA and NA, respectively. During the NA cruise, we hoisted the net at a lower speed than in the GoA to reduce potential stress for the mesozooplankton. The content of the cod end of the plankton net was transferred into a plankton splitter, concentrated over a 63 μm Nitex screen and then placed in 0.2 μm filtered seawater collected at the same location.

**Table 1:** Community‐specific taurine release rates (RR_C_) per individual and per gram C‐biomass of the mixed zooplankton communities of the Gulf of Alaska (GoA) and the North Atlantic (NA) and the percentage of the dominant species in the zooplankton community. Date (month/day), time, depth and number of tows (*N*_tows_) are indicated. Abbreviations: *N. cristatus*, *Neocalanus cristatus*; R, replicates (A, B, C); *R* ^2^, correlation coefficient; *N*_total,_ total number of zooplankton individuals used in each incubation chamber; %, number of individuals of the identified species in relation to the total number of individuals used in the experiment. *T*_I_ is the duration of each experiment and *T*_C_ is the time span used to calculate the release rates from the linear regressions. Incubation temperature was 10°C.

Cruise	Station	R	*T*_C_(h)	*T*_I_(h)	*R* ^2^	*N. cristatus*(%)	*Themisto* sp.(%)	*Vibilia* sp.(%)	N_total_	RR_C_(pmol individual^−1^ h^−1^)	RR_C_(μmol g^−1^ Ch^−1^)	Date	Time (hh : mm)	Depth (m)	*N*_tows_
GoA	5	A	24	24	0.970	32	26	42	50	316.8	0.9	8/8	05:30	600	2
GoA		B	24	24	0.907	51	22	28	83	480.2	0.5	8/8	05:30	600	2
GoA	8		8	24	0.984	0	63	37	38	570.8	1.7	8/9	14:00	600	1
GoA	12		8	24	0.955	59	35	6	51	331.8	0.5	8/11	12:45	600	1
GoA	17		10	24	0.977	79	19	2	53	356.0	0.7	8/13	15:50	600	1
GoA	20		8	24	0.998	78	19	4	54	498.3	0.6	8/15	12:40	600	1
GoA	29		8	24	0.856	56	18	26	39	421.8	0.6	8/18	10:00	600	1
						*Acartia* sp.	*Calanus* sp.	*Centropagus* sp.							
NA	14	A	3	24	0.814	14	45	41	44	24.7	8.9	8/8	12:30	162	1
		B	3	24	0.851	20	32	49	41	24.5	7.7				
		C	3	24	0.970	20	20	59	49	24.1	11.8				

**Table 2:** Species‐specific taurine release rates (RR_S_) per individual and per C‐biomass of selected copepod species obtained in incubation experiments in the North Atlantic. Date (month/day), time, depth and number of tows (*N*_tows_) are indicated. Abbreviations: R, replicates (A, B, C); *R* ^2^, correlation coefficient; *N*_total,_ total number of zooplankton individuals used in each incubation chamber; *T*_I_, the duration of each experiment and *T*_C_ is the time span used to calculate the release rates from the linear regressions. Incubation temperature was 10°C.

Station	Species	R	*T*_C_(h)	*T*_I_(h)	*R* ^2^	*N*_total_	RR_S_(pmol individual^−1^ h^−1^)	RR_S_(μmol g^−1^ Ch^−1^)	Date	Time (hh : mm)	Depth (m)	*N*_tows_
111	*Calanus* sp.	A	8	8	0.807	30	17.9	7.6	8/12	13:25	157	1
		B	8	8	0.772	30	6.9	4.4				
		C	8	8	0.851	35	16.7	7.0				
59	*Acartia* sp.	A	5	8	0.877	37	7.9	3.4	8/19	10:10	205	2
		B	5	8	0.878	35	7.2	4.6				
		C	5	8	0.949	35	7.9	3.2				
115	*Clausocalanus* sp.	A	5	8	0.616	35	5.5	3.8	8/14	16:00	203	2
		B	5	8	0.817	35	4.7	3.3				
		C	5	8	0.740	35	7.1	5.1				
111	*Centropagus* sp.	A	8	8	0.868	30	3.8	1.1	8/12	13:25	157	1
		B	8	8	0.841	30	4.5	1.8				
		C	8	8	0.915	30	3.8	1.1				

Sorting of individual crustacean mesozooplankton species for the Tau release experiments in the NA was done under a dissecting microscope. During sorting and transfer into the incubation vessels care was taken that mesozooplankton specimens were never exposed to air. Specimens were incubated in 500 mL of 0.2 μm filtered seawater (collected at the same location as the zooplankton) in pre‐combusted glass jars under dim light conditions at 10°C. The integrated mean temperatures for the GoA (0–600 m) and for the NA (0–200 m) water column were ∼10°C and ∼15°C, respectively. However, the zooplankton individuals in the NA migrate during the day to depths below 200 m, where they are also exposed to temperatures of ∼10°C. The incubations were conducted at 10°C in both cruises to minimize differences in release rates due to temperature. At each sampling, 4 mL of water was collected for dissolved free Tau and DFAA measurements and 1.5 mL for bacterial abundance. Each of the parameters was sampled in duplicate. Water samples for dissolved free Tau and DFAA measurements were taken at 2 h intervals until 12 h, and at a final time point after 24 h for the incubations in the GoA. During the NA cruise, incubations were conducted in triplicate. Water samples were collected at 0.5 h intervals until 2 h after starting the incubation and, subsequently, at 3 h, 5 h, and 8 h. Additionally, 0.2 μm filtered seawater without mesozooplankton added served as a control (1 per experiment) and was sampled at the same time intervals as the mesozooplankton incubations. Release rates were calculated by linear regression analyses until the time point when the first amino acid species or Tau did not further increase in concentration.

The numbers of mesozooplankton specimens used in each of the incubations are given in Tables 1 and 2. The release rates of dissolved free Tau and DFAA were estimated by least‐square linear regression analysis between the dissolved free Tau or DFAA concentration and the incubation time. Examples for the changes in dissolved free Tau concentrations over time in the mesozooplankton incubations from the GoA and the NA, respectively, are given in Supporting Information Fig. S2.

Release rates (μmol g^−1^ C‐biomass h^−1^) of dissolved free Tau and DFAA species and the ratio of dissolved free Tau to the sum of DFAA released by crustacean zooplankton were also calculated (Supporting Information Table S3). The C‐biomass of the zooplankton species was determined as described below. No release rates were calculated for dissolved free Tau or DFAA species when the regression analyses of the linear increase in dissolved free Tau or DFAA concentration over time in the incubations resulted in an *R* ^2^ < 0.60 and a *p* value > 0.05, or when dissolved free Tau and DFAA were released at the beginning of the experiment and remained constant or decreased thereafter. *R* ^2^ and *p* values are given in Supporting Information Table S4. Asparagine and serine were occasionally coeluting, thus their concentrations are given as sum of Asn + Ser.

### Determining microbial abundance in the mesozooplankton incubations

Microbial abundance was monitored over the course of the mesozooplankton incubation experiments. Water from the incubations (1.5 mL) was fixed with glutaraldehyde (0.5% final concentration) at room temperature for 10 min. Subsequently, the samples were frozen in liquid nitrogen for 10 min and stored at −80°C. Prior to flow cytometric analysis, the samples were thawed to room temperature and 0.5 mL subsamples were stained with SYBR Green I (1× final concentration) in the dark for 10 min, and 1 μm fluorescent beads (Molecular Probes, 1 × 10^5^ mL^−1^) were added to the samples as an internal standard. Prokaryotes were enumerated on an Accuri C6 (Becton Dickinson) based on their signature in a plot of green fluorescence vs. side scatter. No significant increase in microbial abundance over time was observed in the incubation experiments (data not shown).

### Determining the biovolume of the crustacean mesozooplankton used in the incubation experiments

After the incubations, the mesozooplankton specimens were collected, fixed with formaldehyde (4% final concentration) and stored at 4°C until further measurements in the home lab. The length and diameter of the zooplankton specimens were measured under the dissecting microscope with a calibrated Stereo Lumar V.12 (ZEN software) to estimate their biovolume (Supporting Information Table S5). We used the formula of an ellipsoid (*V* = $\frac{4}{3}\pi abc$) (Lawrence et al. 1987) to estimate the biovolume of copepods, and for amphipods we assumed a conical shape (*V* = $\frac{1}{3}\pi r^{2}h$) (Halliday 2001). The biovolume was converted into carbon biomass using 0.08 pg C μm^−3^ for copepods (Beers and Stewart 1970; Monti and Umani 2000), and 0.05 pg C μm^−3^ for amphipods (Mullin 1969).

To estimate the in situ bulk release rates and turnover times of dissolved free Tau (i.e., the time that mesozooplankton would need to release an equal amount of Tau as the measured concentrations in the environment) in the surface waters at both oceanic sites, the mean release rates of dissolved free Tau determined in the incubation experiments and the mean dissolved free Tau concentrations of the upper water column were used along with published copepod abundance data (Supporting Information Table S6) from the northeast Atlantic (Fernández de Puelles et al. 1996) and the western subarctic Pacific (Yamaguchi et al. 2002). The in situ bulk release rates and turnover times were calculated as:

RRt=RRc×A

T=C/RRt

where RRt is the release rate for the whole copepod community (nmol L^−1^ d^−1^), RRc is the mean community‐specific dissolved free Tau release rate obtained in the incubation experiments and given in Table 1 (for the GoA: 425.1 ± 96 pmol individual^−1^ h^−1^ or 10.2 nmol individual^−1^ d^−1^, *n* = 7; for the NA: 24.4 ± 0.3 pmol individual^−1^ h^−1^ or 0.6 nmol individual^−1^ d^−1^, *n* = 3; *A* is the copepod abundance obtained from the literature (number L^−1^), *T* is the turnover time in days (d) and *C* is the mean dissolved free Tau concentration obtained in this study (upper 100 m: GoA: 2.5 nM; NA: 2.6 nM). The turnover times of dissolved free Tau given below have to be considered as estimates due to the potential errors associated with the use of published abundance and biomass data of zooplankton and due to the patchiness of zooplankton. Future research will focus on direct measurements of the turnover time by marine microorganisms.

### Statistical analysis

Statistical analyses (Mann–Whitney Test) were performed with SPSS Statistics 20. The effect size (Hudges *g*) was calculated in Excel. The range of *g* is 0–1 and indicates the effect size between the experiments: *g* < 0.2 indicates small effects, *g* > 0.5 medium effects, and *g* > 0.8 strong effects.

## Results

### Physico‐chemical characteristics of the water column at the respective sites

Potential temperature/salinity diagrams for the study sites are given in Supporting Information Fig. S3. Temperatures in surface waters varied between 14°C and 16°C in the GoA and between 20°C and 24°C in the NA, decreasing with depth until reaching approximately 1.0°C and 2.5°C in the deepest layers of the GoA and NA, respectively. Salinity generally increased with depth in the GoA with lowest values in surface water (∼31.5) and maxima in the deep ocean (∼34.7). In the NA, low salinity was recorded in surface waters (34.5) and below 1200 m depth (35.5), corresponding to Labrador Seawater and North Atlantic Deep Water (van Aken 2007). The highest salinity (∼36) at the NA site was measured at around 1000 m depth, corresponding to Mediterranean Water.

Dissolved oxygen concentrations were generally lower in the GoA than in the NA (Supporting Information Fig. S4) except in surface waters. The oxygen minimum was located at ∼1000 m depth at both sampling areas, however it was much more pronounced in the GoA, where suboxic levels were detected, than in the NA.

### Dissolved free Tau concentrations throughout the water column

**Figure 2:**
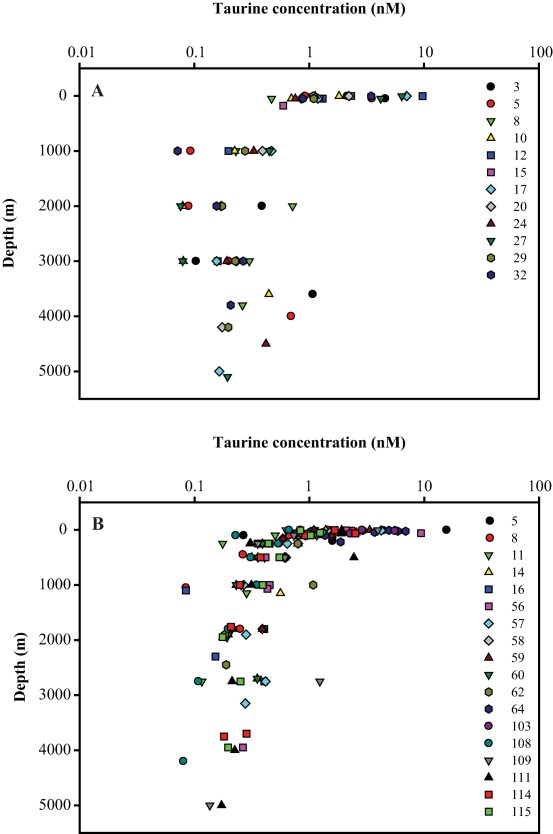
Depth profiles of dissolved free taurine concentrations in (**A**) the Gulf of Alaska (GoA) and (**B**) the North Atlantic (NA). Stations are indicated by symbols and colors.

Dissolved free Tau concentrations were in the 1–16 nM range in surface waters and decreased with depth to about 0.1 nM below 200 m in both the GoA and NA (Fig. 2). Thus, dissolved free Tau concentrations were significantly higher in the epipelagic than in the meso‐ and bathypelagic layers at both sites (Mann–Whitney, *p* < 0.05). No significant differences in dissolved free Tau concentrations, however, were obtained between the meso‐ and bathypelagic layers, and between the two sites (Mann–Whitney, *p* > 0.05). In the epipelagic layer of the NA, the highest dissolved free Tau concentration was obtained close to the coast (up to 15.7 nM). High dissolved free Tau concentrations in the GoA were also found at more coastal stations (ranging between 4.2 nM and 9.7 nM) than further offshore (Fig. 2).

The contribution of dissolved free Tau‐C to DOC was generally low. In the upper epipelagic realm it ranged from ∼0.001% to 0.1% at both oceanic sites, while in the meso and bathypelagic layers it was 0.0001–0.01% (Supporting Information Fig. S5A). The contribution of dissolved free Tau‐N to DON in the GoA was about one order of magnitude higher than the contribution of dissolved free Tau‐C to the DOC pool (Supporting Information Fig. S5).

### Dissolved free Tau release by crustacean mesozooplankton

**Figure 3:**
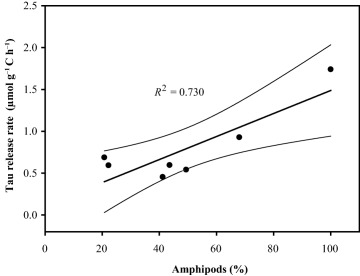
Relation between taurine release rates (in μmol g^−1^ C‐biomass h^−1^) of crustacean mesozooplankton assemblages and the contribution of amphipods to the mesozooplankton assemblage in the Gulf of Alaska. *R* ^2^ and confidence intervals (95%) are indicated.

In the GoA, the dominant mesozooplankton species were the calanoid copepod *Neocalanus cristatus* and the amphipods *Themisto* sp. and *Vibilia* sp. (Table 1). The community‐specific dissolved free Tau release rates ranged between 0.5 μmol g^−1^ C‐biomass h^−1^ and 1.7 μmol g^−1^ C‐biomass h^−1^. A positive relationship was observed between dissolved free Tau release rates and the contribution of amphipods to the zooplankton assemblage (*R* ^2^ = 0.730; Fig. 3). However, the sample with 100% contribution of amphipods is strongly influencing this relationship. In the GoA, the release rates per mesozooplankton specimen ranged between 317 pmol individual^−1^ h^−1^ and 571 pmol individual^−1^ h^−1^ (Table 1). The highest community‐specific dissolved free Tau release rates per individual as well as per biomass in the GoA were obtained for an assemblage consisting exclusively of amphipods (Table 1).

The mesozooplankton community collected in the GoA exhibited higher dissolved free Tau release rates per specimen than in the NA (Table 1). Community‐specific dissolved free Tau release rates normalized to zooplankton biomass, however, were about one order of magnitude higher in the NA than in the GoA (Mann–Whitney, *p* < 0.05; *g* > 0.9; Table 1). Thus, the large‐sized zooplankton of the GoA exhibited lower dissolved free Tau release rates per biomass than the small‐sized zooplankton of the NA (Supporting Information Fig. S6).

In the NA, the community‐specific dissolved free Tau release rates obtained from the mixed copepod community were higher than the species‐specific release rates obtained in the single‐species incubations (compare RR_C_ and RR_S_ in Tables 1 and 2, respectively). The community‐specific dissolved free Tau release rates with *Calanus* sp., *Acartia* sp. and *Centropagus* sp. dominating the mixed community ranged between 7.7 μmol g^−1^ C‐biomass h^−1^ and 11.8 μmol g^−1^ C‐biomass h^−1^ (Table 1). In contrast, species‐specific dissolved free Tau release rates ranged from 1.1 μmol g^−1^ C‐biomass h^−1^ to 7.6 μmol g^−1^ C‐biomass h^−1^ (Table 2, Supporting Information Fig. S6). The effect size (Supporting Information Table S7) varied from 0.6 (*Calanus* sp.) to 0.9 (*Centropagus* sp.), indicating an inherent difference between the community‐specific and the species‐specific dissolved free Tau release rates. *Calanus* sp. exhibited significantly higher species‐specific dissolved free Tau release rates (4.4–7.6 μmol g^−1^ C‐biomass h^−1^; Table 2; Supporting Information Fig. S6) than *Centropagus* sp. (1.1–1.8 μmol g^−1^ C‐biomass h^−1^; *p* < 0.05, *g* = 0.9). Similar species‐specific dissolved free Tau release rates were measured for *Clausocalanus* sp. (3.3–5.1 μmol g^−1^ C‐biomass h^−1^) and *Acartia* sp. (3.2–4.6 μmol g^−1^ C‐biomass h^−1^).

### Estimating dissolved free Tau release rates and turnover times in the water column

Since copepods account for > 90% of total abundance of mesozooplankton (Longhurst 1985) in the world's oceans, we used copepod abundance data from the literature (Supporting Information Table S6), the mean community‐specific dissolved free Tau release rates and mean dissolved free Tau concentrations obtained in this study to estimate bulk dissolved free Tau release rates and turnover times in the water column. We assumed steady state in situ dissolved free Tau concentrations. In epipelagic waters (0–100 m depth) the integrated mean dissolved free Tau concentration was 2.5 nM in the GoA and 2.6 nM in the NA. Given the range of copepod abundance for the western subarctic Pacific and the northeast Atlantic (Supporting Information Table S6) and the mean community‐specific dissolved free Tau release rates (*see* Table 1) of 10.2 nmol individual^−1^ d^−1^ and 0.58 nmol individual^−1^ d^−1^ for the GoA and the NA, respectively, we obtain a bulk dissolved free Tau release rate of the copepod community of 35.8–51.9 nmol L^−1^ d^−1^ and 1.1–2.6 nmol L^−1^ d^−1^ for the GoA and the NA epipelagic waters, respectively. Based on the bulk dissolved free Tau release rates and the dissolved free Tau concentrations, we estimate Tau turnover times of 0.05–0.07 d and 1.0–2.3 d for the epipelagic waters of the GoA and the NA, respectively.

### DFAA released by crustacean zooplankton

Glycine was the main DFAA species released by zooplankton in almost all the release experiments (Supporting Information Table S3). In the GoA, glycine, arginine, and alanine were the main DFAA species released by zooplankton, while threonine, arginine, histidine, and alanine, among others, varied in their contribution to the total DFAA pool released by zooplankton in the NA. The release ratios of dissolved free Tau to total DFAA (mol/mol) ranged between 0.06–0.28 in the GoA and 0.01–0.4 in the NA (Supporting Information Table S3).

## Discussion

### Zooplankton as a source of dissolved free Tau in the open ocean

Generally, the depth profiles of dissolved free Tau concentrations in the Pacific and Atlantic (Fig. 2) are similar to the more commonly reported depth distribution of DFAA. The low and fairly uniform dissolved free Tau concentrations in epipelagic waters might be linked to an efficient uptake by heterotrophic bacteria as reported for DFAA (Fuhrman 1987; Keil and Kirchman 1999; Zubkov et al. 2008). Also, the low contributions of dissolved free Tau‐C to DOC and Tau‐N to DON throughout the water column support the conclusion of its rapid turnover (Supporting Information Fig. S5). The vertical decrease of dissolved free Tau concentrations (Fig. 2) and the low contribution of dissolved free Tau‐C to DOC from the epipelagic to the mesopelagic waters (Supporting Information Fig. S5) probably reflect the lower zooplankton metabolic activity associated with the lower temperatures, the lower zooplankton biomass in meso and bathypelagic waters (Yamaguchi et al. 2002) and depth‐related differences in taxonomic zooplankton composition (Coyle and Pinchuk 2005; Vereshchaka et al. 2016), resulting, overall, in lower dissolved free Tau release rates in deep waters.

Copepods dominate mesozooplankton communities over a wide variety of ecological conditions in the ocean. In general, excretion rates by zooplankton vary depending on the physiological state of the organisms, which varies among oceanic provinces and seasons and zooplankton characteristics (Conover 1959, 1968; Kawall et al. 2001; Helland et al. 2003). According to Webb and Johannes (1967), copepod‐dominated zooplankton assemblages exhibit higher dissolved free Tau release rates than chaetognath‐ or scyphozoan‐dominated zooplankton, emphasizing the importance of crustacean zooplankton as a source of dissolved free Tau for the epipelagic and upper mesopelagic water column. Besides dissolved free Tau, the main amino acids released by crustacean zooplankton in the GoA were glycine, arginine, and alanine (Supporting Information Table S3), which are also the main constituents of the free amino acid pool of many crustaceans such as copepods and krill (Srinivasagam et al. 1971; van der Meeren et al. 2008). The large Pacific zooplankton specimens exhibit lower dissolved free Tau (Tables 1 and 2) and DFAA (Supporting Information Table S3) release rates per unit biomass, reflecting the general inverse relationship of biovolume‐normalized excretion rates and body size (Wen and Peters 1994; Hall et al. 2007). Consequently, lower Tau inputs in the deep ocean could also be linked to lower excretion rates associated with macro‐ (e.g., shrimps) and larger mesozooplankton (e.g., copepods, amphipods) dominating the meso‐ and bathypelagic zooplankton biomass (Coyle and Pinchuk 2005; Vereshchaka et al. 2016). Excretion rates and the element ratios of organic compounds released by zooplankton can vary strongly depending on the feeding strategy and behavior and food quality and quantity (Kleppel 1993; Kiørboe et al. 1996; Frangoulis et al. 2004). The higher release rates of dissolved free Tau obtained in experiments with an increasing contribution of amphipods (Fig. 3) might be due to the amphipods' carnivorous diet (Saba et al. 2009). Future studies should include more incubation with a higher percentage of carnivore species, which expectedly should most likely result in a stronger relationship.

Mixed copepod communities and *Calanus* sp. incubations resulted in higher dissolved free Tau release rates than incubations with single species in the NA (Tables 1 and 2, Supporting Information Fig. S6). This outcome might be linked to the ability of some copepod species to switch to other prey items (Metz and Schnack‐Schiel 1995; Kiørboe et al. 1996); consequently, food quality and quantity might have varied for the individual zooplankton species.

The nutritive state, sex, and developmental stage of different zooplankton species might cause intraspecific variations in their dissolved free Tau and DFAA release patterns (Mitra and Flynn 2007; Saage et al. 2009; Almeda et al. 2011). The vertical tows were obtained at different times of the day (Tables 1 and 2) and from different depths (down to ∼600 m in GoA and ∼200 m in NA), so the mesozooplankton collected could have been in different nutritive states. In general, intraspecific variations in dissolved free Tau release patterns were low with the exception of *Calanus* sp. (Table 2), in contrast to the DFAA release patterns (Supporting Information Table S3) that were more variable.

The high concentrations of DFAA and dissolved free Tau detected at the beginning of some experiments were probably caused by stress induced by the collection (e.g., net tow) and handling (e.g., separation of individuals) of the organisms. Also, the use of a higher towing speed during the GoA cruise (30 m min^−1^) might have led to an enhanced excretion rate at the beginning of the incubations than in the NA (15 m min^−1^), although care was taken to minimize disturbance. In contrast, starvation could lead to an underestimation of the actual dissolved free Tau release rates. It is well known that DFAA release rates of copepods are much lower when starved (Fuhrman 1987). Although 0.2 μm filtered seawater was used in the release experiments, it is likely that bacteria attached to the carapace of the mesozooplankton took up a fraction of the dissolved free Tau and DFAA released by zooplankton (Carman and Dobbs 1997; Tang et al. 2010). Consequently, our reported dissolved free Tau and DFAA release rates likely resemble more closely “net” rather than “gross” release rates.

Our estimated dissolved free Tau turnover times (0.05–2.3 d) for the epipelagic layer are similar to reported DFAA turnover times, ranging from minutes to a few days (Sipler and Bronk 2015). Nevertheless, our estimates of Tau turnover times are likely conservative, particularly in the epipelagic waters, as we did not include other potentially important sources of Tau such as algae. Also, zooplankton excretion rates are often linearly related to temperature, adding to uncertainty in our estimates on dissolved free Tau turnover times (Webb and Johannes 1967; Chen and Lai 1993). Enhanced excretion rates with increasing temperatures can be associated with either higher ingestions rates (Kiørboe et al. 1982) or stress (Bayne 1973; Bayne and Scullard 1977). Consequently, an incubation temperature higher than in situ might result in higher release rates and consequently in lower turnover times in the warmer NA (20–24°C) than in the GOA (14–16°C). But in order to minimize this effect, and considering that zooplankton migrate to deeper layers with lower temperatures, all the incubations were performed at 10°C.

The situation in deep waters is generally more complex as zooplankton are present in lower abundance than in epipelagic waters, showing a patchy and depth‐dependent distribution (Vereshchaka et al. 2016). In this study we focused on concentrations and the direct release of dissolved free Tau, but Tau can also occur conjugated with a variety of organic acids. For example, Tau‐bearing lipids were found in the metabolites and release products of various copepod genera, which tend to accumulate these compounds under starvation (Mayor et al. 2015; Selander et al. 2015, 2016). Consequently, it is reasonable to assume that in the deep ocean, where food is limited and temperature low, zooplankton accumulate dissolved free Tau and Tau‐conjugated compounds. A large number of copepod species (e.g., *Neocalanus* sp., *Calanus* sp.) exhibits vertical migration into mesopelagic and bathypelagic waters for hibernation every year (Jónasdóttir et al. 2015), with mortality rates up to 75% (Longhurst and Williams 1992; Zhang and Dam 1997). Thus, it is possible that copepod carcasses and their fecal pellets (via leaching and decay processes) are the predominant sources of dissolved free Tau and other compounds for deep‐sea microbes, rather than active release by zooplankton.

The dissolved free Tau release rates obtained in this study and the low dissolved free Tau turnover times in epipelagic waters indicate that dissolved free Tau might be an important substrate for heterotrophic microbes, a conclusion supported by several recent meta‐genomics and ‐proteomics (Sowell et al. 2009, 2011; Williams et al. 2012; Wilkins et al. 2013), as well as by experimental studies (Schwalbach et al. 2010; Steindler et al. 2011).

Our results show that crustacean zooplankton release copious amounts of dissolved free Tau, especially in epipelagic and upper mesopelagic waters. There is growing evidence of the potential significance of this compound for marine microbes. Tau transporters and enzymes involved in Tau utilization are widespread among diverse bacterial and archaeal phylotypes (e.g., Walker et al. 2010; Williams and Cavicchioli 2014) throughout the ocean water column (e.g., Smith et al. 2013; Hawley et al. 2014; León‐Zayas et al. 2015). Members of SAR11, the most abundant bacterial clade in the oceans, require exogenous reduced sulfur due to their deficiency in assimilatory sulfate reduction genes (Tripp et al. 2008) and can effectively grow on Tau (Steindler et al. 2011). The short turnover times of dissolved free Tau are similar to those reported for DFAA, supporting the notion that dissolved free Tau represents an important substrate and energy source for heterotrophic microbes, particularly in the epipelagic and mesopelagic waters. Hence, there is a metabolic link between zooplankton activity and its associated Tau release and heterotrophic bacteria in the ocean.

## Conflict of Interest

None declared.

## Supporting information

Supporting Information Figure 1.

Supporting Information Figure 2.

Supporting Information Figure 3.

Supporting Information Figure 4.

Supporting Information Figure 5.

Supporting Information Figure 6.

Supporting Information Tables.
