# Supplementary material for: Crustacean zooplankton release copious amounts of dissolved organic matter as taurine in the ocean
Source: Limnol Oceanogr. 2017 Jun 20;62(6):2745–58. doi: 10.1002/lno.10603 (PMC5724677; doi:10.1002/lno.10603)
Supplement: Supplementary file 1 — Supporting Information Figure 1. [file LNO-62-2745-s001.pdf]

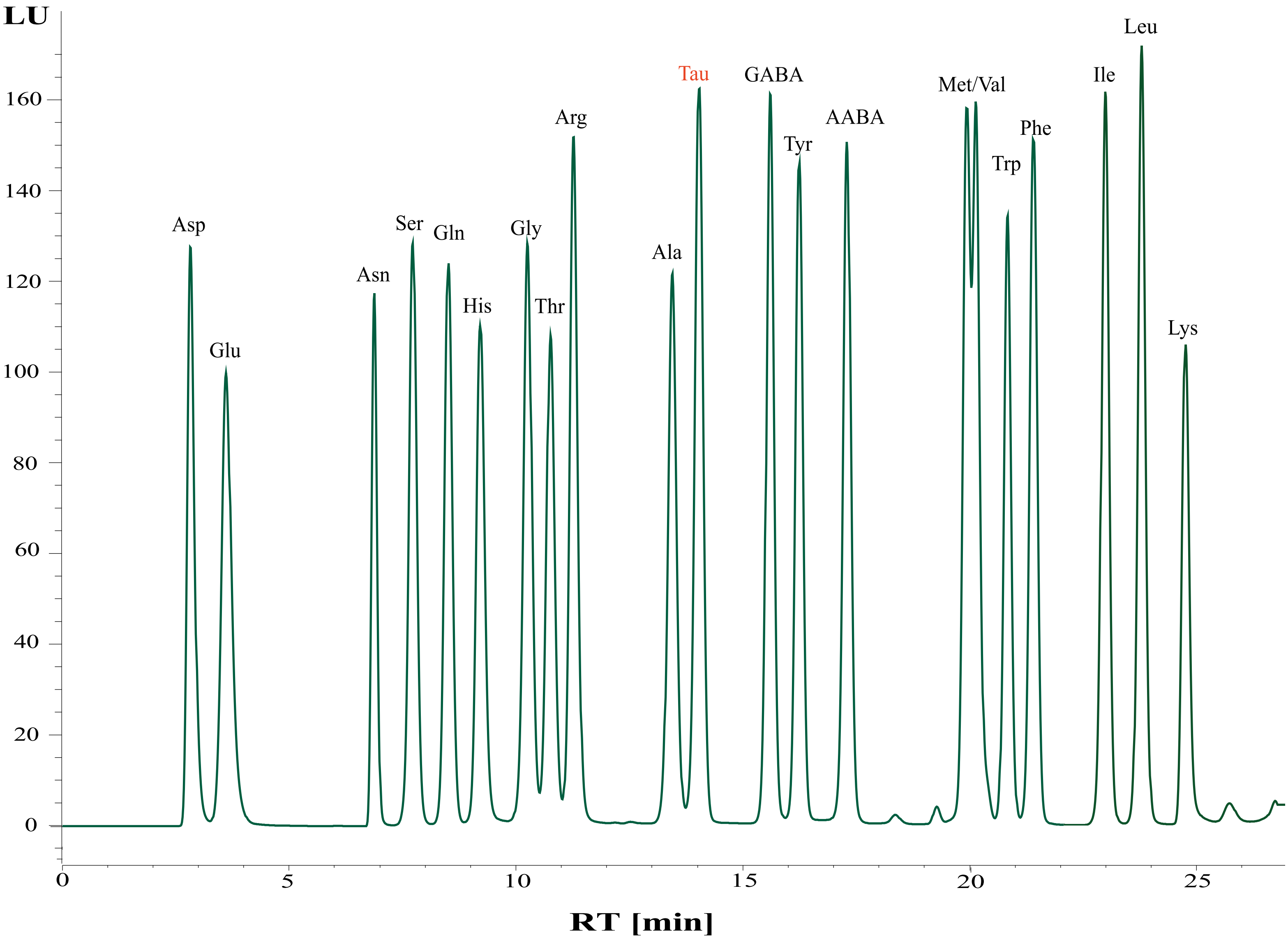

Supplementary Figure 1. Example of a chromatogram showing the separation of amino acids standards (1uM, Gain 10). Abbreviations: RT, retention time; LU, light units; Asp, Asparagine acid; Glu, Glutmaric acid; Asn, Asparagine; Ser, Serine; Glu, Glutamine; His, Histine; Gly, Glycine; Arg, Arginine; Thr, Threonine; Ala, Alanine; Tau, Taurine; GABA, gamma-aminobutyric acid; Tyr, Tyrosine; AABA, alpha-aminobutyric acid; Met, Methionine; Val, Valine; Trp, Tryptophane; Phe, Phenylalanine; Ile, Isoleucine; Leu, Leucine; Lys, Lysine.
