# Supplementary material for: Crustacean zooplankton release copious amounts of dissolved organic matter as taurine in the ocean
Source: Limnol Oceanogr. 2017 Jun 20;62(6):2745–58. doi: 10.1002/lno.10603 (PMC5724677; doi:10.1002/lno.10603)
Supplement: Supplementary file 2 — Supporting Information Figure 2. [file LNO-62-2745-s002.pdf]

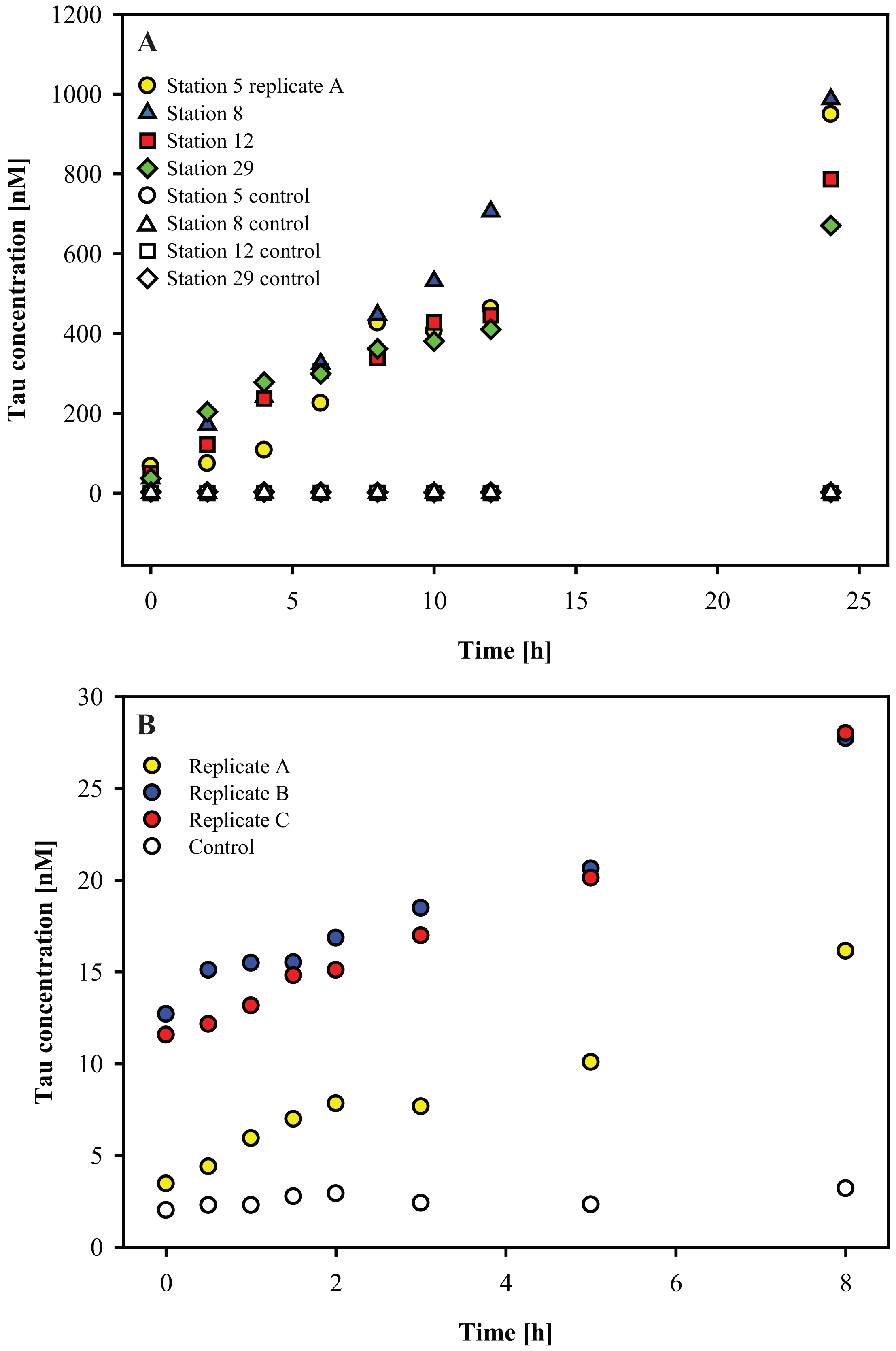

Supplementary Figure 2: Examples of the taurine release over time: crustacean mesozooplankton community of the Gulf of Alaska - stations 5 (replicate A), 8, 12, 29 and the controls (A), mixed copepod community of the North Atlantic in triplicate and the control (B).
