# Supplementary material for: Crustacean zooplankton release copious amounts of dissolved organic matter as taurine in the ocean
Source: Limnol Oceanogr. 2017 Jun 20;62(6):2745–58. doi: 10.1002/lno.10603 (PMC5724677; doi:10.1002/lno.10603)
Supplement: Supplementary file 3 — Supporting Information Figure 3. [file LNO-62-2745-s003.pdf]

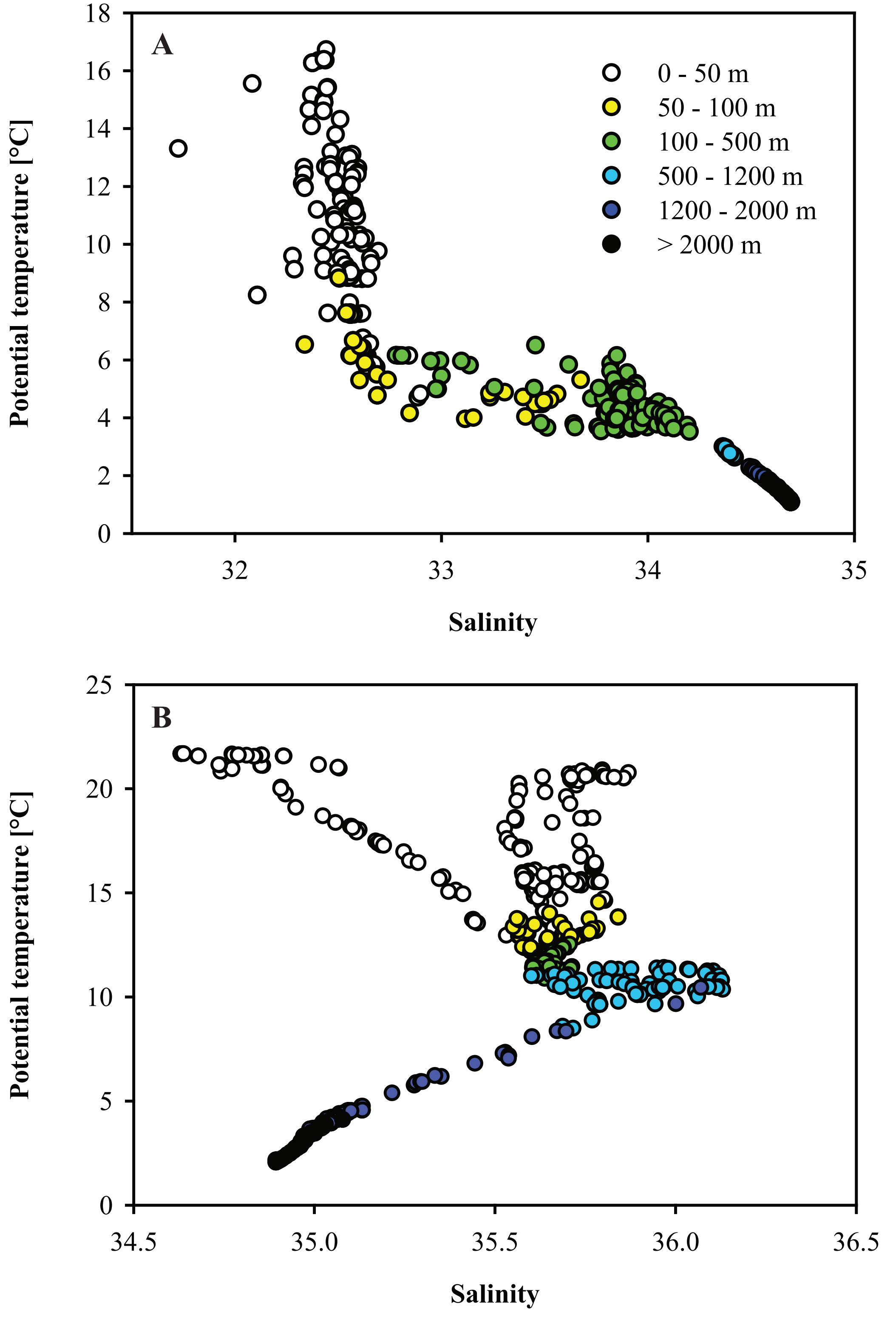

Supplementary Figure 3. Temperature/salinity diagrams of the waters collected (A) in the Gulf of Alaska and (B) in the North Atlantic. Different depth layers are indicated by different colors.
