# Supplementary material for: Crustacean zooplankton release copious amounts of dissolved organic matter as taurine in the ocean
Source: Limnol Oceanogr. 2017 Jun 20;62(6):2745–58. doi: 10.1002/lno.10603 (PMC5724677; doi:10.1002/lno.10603)
Supplement: Supplementary file 4 — Supporting Information Figure 4. [file LNO-62-2745-s004.pdf]

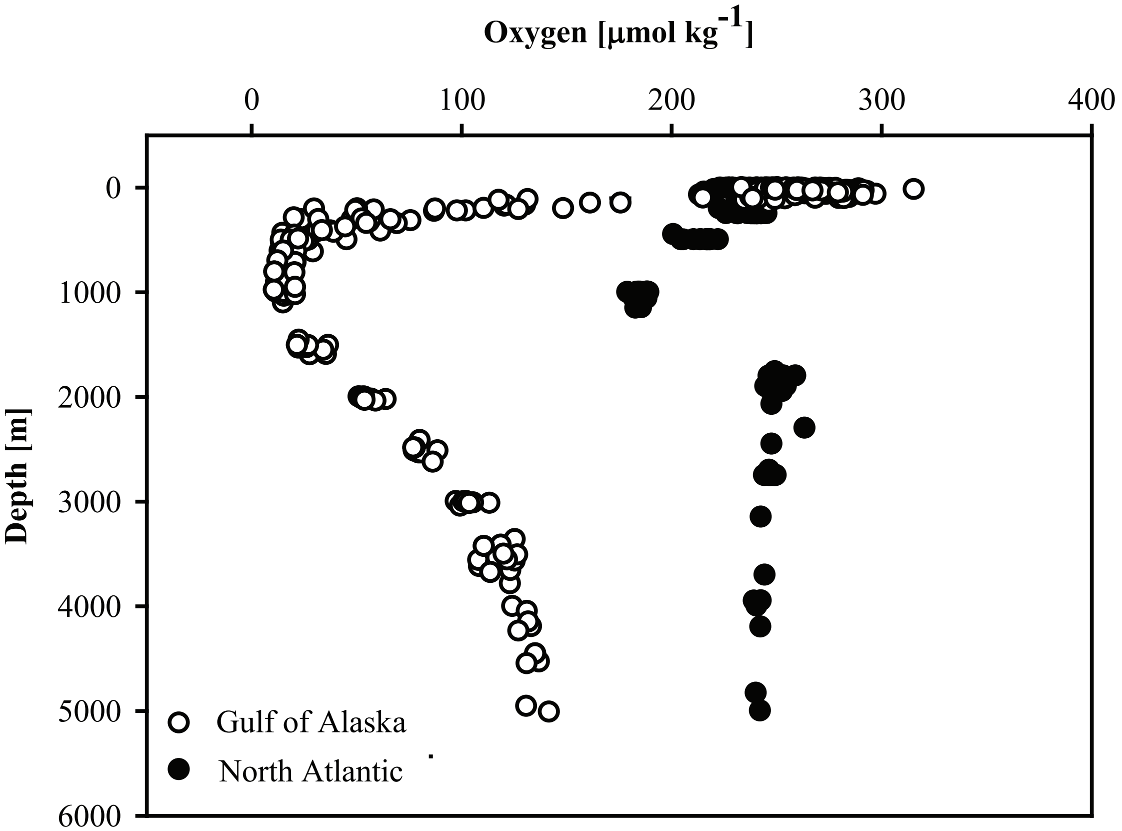

Supplementary Figure 4. Depth profiles of dissolved oxygen concentrations in the Gulf of Alaska (empty circles) and North Atlantic (filled circles).
