# Supplementary material for: Crustacean zooplankton release copious amounts of dissolved organic matter as taurine in the ocean
Source: Limnol Oceanogr. 2017 Jun 20;62(6):2745–58. doi: 10.1002/lno.10603 (PMC5724677; doi:10.1002/lno.10603)
Supplement: Supplementary file 5 — Supporting Information Figure 5. [file LNO-62-2745-s005.pdf]

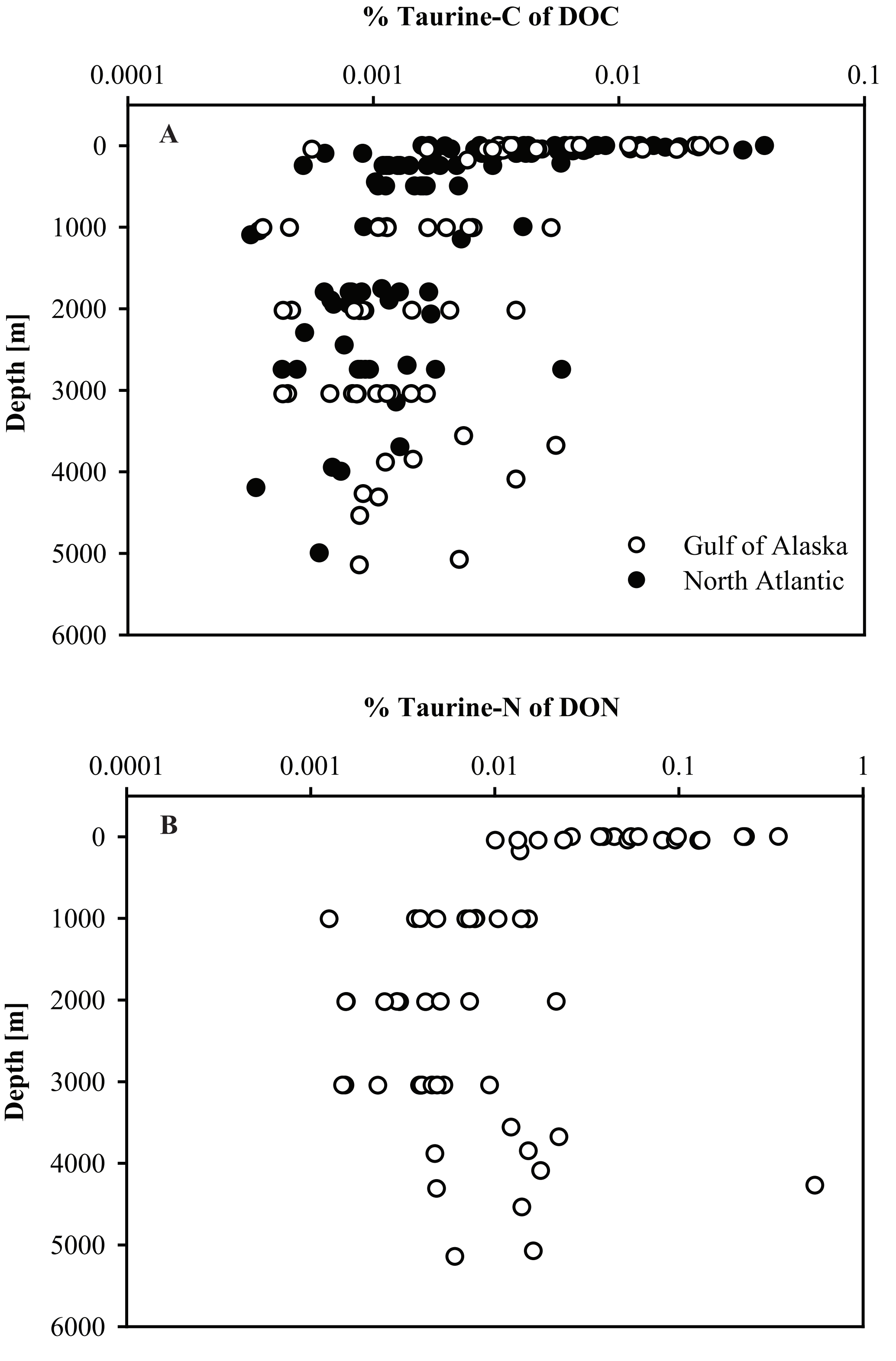

Supplementary Figure 5. Depth profiles of the contribution of taurine-C to the DOC pool (%) in the Gulf of Alaska and North Atlantic (A) and of the taurine-N to the DON pool (%) in the Gulf of Alaska (B). DON data for the North Atlantic were not determined. Abbreviations: DOC, dissolved organic carbon; DON, dissolved organic nitrogen.
