# Supplementary material for: Crustacean zooplankton release copious amounts of dissolved organic matter as taurine in the ocean
Source: Limnol Oceanogr. 2017 Jun 20;62(6):2745–58. doi: 10.1002/lno.10603 (PMC5724677; doi:10.1002/lno.10603)
Supplement: Supplementary file 6 — Supporting Information Figure 6. [file LNO-62-2745-s006.pdf]

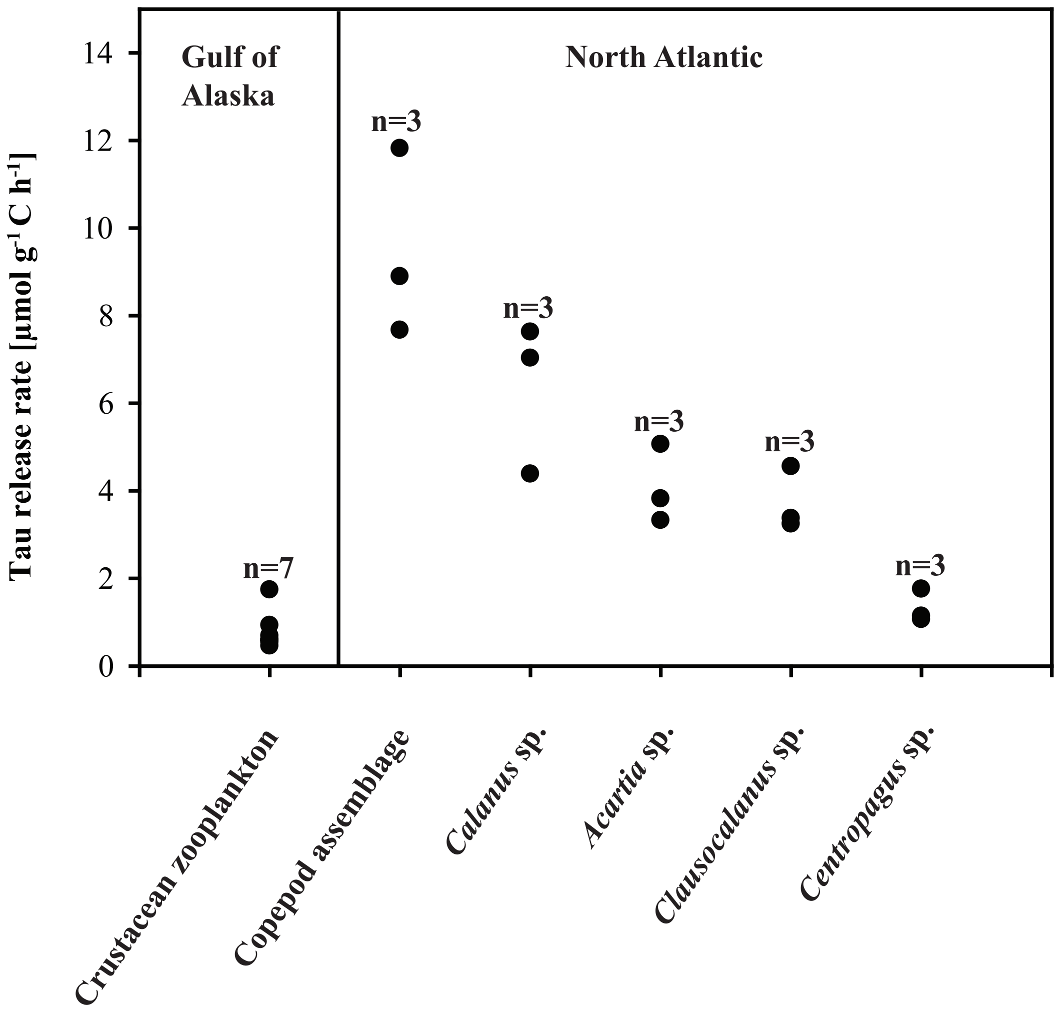

Supplementary Figure 6. Taurine release rates (in µmol g^{-1} C-biomass h^{-1}) from different copepod species and mixed communities in the North Atlantic and from amphipod-copepod assemblages in the Gulf of Alaska.
