# Supplementary material for: Crustacean zooplankton release copious amounts of dissolved organic matter as taurine in the ocean
Source: Limnol Oceanogr. 2017 Jun 20;62(6):2745–58. doi: 10.1002/lno.10603 (PMC5724677; doi:10.1002/lno.10603)
Supplement: Supplementary file 7 — Supporting Information Tables. [file LNO-62-2745-s007.docx]

**Supplementary Table 1.** Elution gradient used for the separation of dissolved free taurine and free amino acids by HPLC. Eluent A: 50 mM sodium acetate buffer (pH = 6.8); B: methanol; C: tetrahydrofuran.

| **Elution time [min]** | **Eluent [%]** | | |
| --- | --- | --- | --- |
|  | **A** | **B** | **C** |
| 0.0 | 90.0 | 10.0 | 0.0 |
| 2.0 | 90.0 | 7.4 | 2.6 |
| 22.3 | 40.0 | 59.0 | 1.0 |
| 23.5 | 25.0 | 75.0 | 0.0 |
| 24.5 | 25.0 | 75.0 | 0.0 |
| 25.0 | 90.0 | 10.0 | 0.0 |
| 28.0 | 90.0 | 10.0 | 0.0 |

**Supplementary Table 2.** Parameters for validation of the HPLC method used to measure dissolved free taurine concentration in seawater. Abbreviations: *R^2^*, correlation coefficient; LOD, limit of detection; LOQ, limit of quantification; RSD, relative standard deviation; AABA, α-aminobuturyic acid; (-) non-spiked taurine; (+) spiked with 1 nM taurine. **^a^** seawater samples were measured with a gain factor of 14 and samples from the zooplankton release experiment with a gain factor of 10 or 12 depending on the taurine concentrations. **^b^** the ranges are given for *R^2^*, LOD and LOQ (depending on analytical conditions: e.g. column age).

| **Substance** | **Linearity^a^** | ***R^2^*^b^** | **LOD^b^** | **LOQ^b^** | **Recovery/RSD (*n* = 5; %)** | | | | | | **Accuracy (*n* = 8)** | |
| --- | --- | --- | --- | --- | --- | --- | --- | --- | --- | --- | --- | --- |
|  | **[nM]** |  | **[nM]** | **[nM]** | **[nM]** | | | | | | **[nM]** | |
|  |  |  |  |  | **1** | **5** | **10** | **500** | **1000** | **1500** | **-** | **+** |
| Taurine | 0.01-3000 | 0.997-0.999 | 0.01-0.02 | 0.1-0.2 | 98.9±0.49 | 100.1±0.18 | 101.8±0.16 | 100.0±0.11 | 99.2±0.07 | 110.6 ± 0.03 | 0.25±0.001 | 1.25±0.003 |
| AABA | 0.01-3000 | 0.998-0.999 | 0.01-0.02 | 0.1-0.2 |  |  |  |  |  |  |  |  |

**Supplementary Table 3A.** Release rates (RR) of dissolved free taurine and free amino acids (DFAA) by crustacean zooplankton in µmol g^-1^ C-biomass h^-1^ in the Gulf of Alaska (GoA). The time in hours (h) used to calculate the linear regression between concentrations of Tau and DFAA species over time for each experiment is given. The ratio of taurine to the total DFAA (mol/mol) released is also indicated. A, B indicate the replicates of each experiment. The red color indicates an *R^2^* > 0.600 and a *p*-value > 0.05 as shown in Table S4. (-) means, it was not possible to calculate a release rate. Abbreviations: St, station; R, replicates; Asp, Aspartic acid; Glu, Glutamic acid; Asn, Asparagine; Ser, Serine; Gln, Glutamine; His, Histine; Gly, Glycine; Arg, Arginine; Thr, Threonine; Ala, Alanine; Tau, Taurine; GABA, gamma aminobutyric acid; Tyr, Tyrosine; Met, Methionine; Val, Valine; Trp, Tryptophan; Phe, Phenylalanine; Ile, Isoleucine; Leu, Leucine; Lys, Lysine.

| **St.** | **R** | **Asp** | **Glu** | **Asn/Ser** | **Asn** | **Ser** | **Gln** | **His** | **Gly** | **Arg** | **Thr** | **Ala** | **Tau** | **GABA** | **Tyr** | **Met/Val** | **Trp** | **Phe** | **Ile** | **Leu** | **Lys** | **Tau/DFAA** | **h** |
| --- | --- | --- | --- | --- | --- | --- | --- | --- | --- | --- | --- | --- | --- | --- | --- | --- | --- | --- | --- | --- | --- | --- | --- |
| 5 | A | 0.3 | 0.2 | - | 0.1 | 0.2 | 0.3 | 0.1 | 1.4 | 0.6 | 1.2 | 0.4 | 0.9 | 0.1 | 0.2 | 0.5 | - | 0.2 | 0.3 | 0.5 | 0.5 | 0.11 | 24 |
| 5 | B | 0.1 | 0.1 | 0.2 | - | - | 0.3 | 0.1 | 0.8 | 0.2 | 0.9 | 0.4 | 0.5 | - | 0.2 | 0.2 | - | 0.4 | 0.2 | 0.4 | 0.5 | 0.10 | 24 |
| 8 | A | 0.6 | 1.5 | - | 1.6 | 0.3 | 0.2 | 0.2 | 2.9 | 0.6 | 0.6 | 1.6 | 1.7 | - | 0.2 | 0.7 | - | 0.6 | 0.3 | 0.5 | 0.6 | 0.12 | 8 |
| 12 | A | 0.1 | 0.4 | 0.2 | - | - | 0.2 | 0.1 | 1.1 | 0.2 | 0.5 | 0.4 | 0.5 | - | 0.1 | 0.2 | - | 0.1 | 0.1 | 0.2 | 0.2 | 0.11 | 8 |
| 17 | A | 0.1 | 0.2 | 0.2 | - | - | - | 0.1 | 1.0 | 0.1 | 0.3 | 0.4 | 0.7 | - | 0.1 | 0.2 | - | - | 0.1 | 0.2 | 0.2 | 0.18 | 10 |
| 20 | A | 0.2 | 0.3 | - | 0.3 | 1.0 | 0.2 | 0.2 | 1.0 | 0.4 | 1.6 | 1.1 | 0.6 | - | 0.3 | 0.5 | - | 0.4 | 0.5 | 0.7 | 1.0 | 0.06 | 8 |
| 29 | A | 0.1 | 0.2 | - | 0.1 | 0.1 | 0.1 | 0.2 | 0.4 | - | - | 0.1 | 0.6 | - | - | 0.1 | - | - | - | - | - | 0.28 | 8 |

**Supplementary Table 3B.** Release rates (RR) of dissolved free taurine and free amino acids (DFAA) by crustacean zooplankton in µmol g^-1^ C-biomass h^-1^ in the North Atlantic (NA). The time in hours (h) used to calculate the linear regression between concentrations of taurine and DFAA species over time for each experiment is given. The ratio of taurine to the total DFAA (mol/mol) released is also indicated. A, B,C indicate the replicates of each experiment. The red color indicates an *R^2^* > 0.600 and a *p*-value > 0.05; orange indicates an *R^2^* < 0.600 and a *p*-value < 0.05 as shown in Table S4. (-) means, it was not possible to calculate a release rate. Abbreviations: MMC, mixed copepod community; Sp, Species; R, Replicate; Asp, Aspartic acid; Glu, Glutamic acid; Asn, Asparagine; Ser, Serine; Gln, Glutamine; His, Histine; Gly, Glycine; Arg, Arginine; Thr, Threonine; Ala, Alanine; Tau, Taurine; GABA, gamma aminobutyric acid; Tyr, Tyrosine; Met, Methionine; Val, Valine; Trp, Tryptophan; Phe, Phenylalanine; Ile, Isoleucine; Leu, Leucine; Lys, Lysine.

| **Sp.** | **R** | **Asp** | **Glu** | **Asn/Ser** | **Asn** | **Ser** | **Gln** | **His** | **Gly** | **Arg** | **Thr** | **Ala** | **Tau** | **GABA** | **Tyr** | **Met/Val** | **Trp** | **Phe** | **Ile** | **Leu** | **Lys** | **Tau/DFAA** | **h** |
| --- | --- | --- | --- | --- | --- | --- | --- | --- | --- | --- | --- | --- | --- | --- | --- | --- | --- | --- | --- | --- | --- | --- | --- |
| MCC | A | 15.2 | 6.8 | 56.7 | - | - | - | 59.8 | 57.4 | 21.7 | 3.9 | - | 8.9 | - | 3.6 | - | - | 4.2 | 3.9 | 5.9 | - | 0.04 | 3 |
| MCC | B | - | 8.5 | - | - | - | - | - | 35.1 | 27.9 | 7.9 | 9.5 | 7.7 | - | - | - | - | - | - | - | - | 0.08 | 3 |
| MCC | C | - | - | - | - | - | - | 6.7 | 61.1 | - | 14.2 | - | 11.8 | - | 2.3 | 4.9 | - | 7.5 | 3.0 | 5.7 | - | 0.11 | 3 |
| *Calanus* sp. | A | - | - | - | - | - | - | - | 7.4 | 2.8 | - | 0.6 | 7.6 | 0.1 | 0.6 | - | - | - | - | - | - | 0.40 | 8 |
| *Calanus* sp. | B | - | - | - | - | - | - | 2.9 | 17.1 | - | 5.6 | - | 4.4 | - | 1.0 | - | - | - | - | 1.9 | 7.4 | 0.11 | 8 |
| *Calanus* sp. | C | - | - | 6.1 | - | - | - | 2.8 | 38.2 | 4.3 | - | - | 7.0 | - | - | - | - | 2.1 | 1.2 | 2.1 | 3.3 | 0.10 | 8 |
| *Acartia* sp. | A | - | - | - | - | - | 2.6 | 0.7 | 4.9 | - | 3.0 | - | 3.4 | - | - | - | - | - | - | - | - | 0.23 | 5 |
| *Acartia* sp. | B | - | - | - | - | - | - | - | 5.7 | - | 6.1 | 6.3 | 4.6 | - | - | - | - | - | - | - | - | 0.20 | 5 |
| *Acartia* sp. | C | - | - | - | - | - | - | - | 15.7 | 7.1 | 3.4 | - | 3.2 | - | - | - | - | - | - | - | 5.2 | 0.09 | 5 |
| *Clausocalanus* sp. | A | - | - | - | - | - | - | - | 3.8 | - | - | 2.6 | 3.8 | - | - | - | - | - | - | - | - | 0.37 | 5 |
| *Clausocalanus* sp. | B | - | - | - | - | - | - | - | 12.4 | 9.1 | - | 7.1 | 3.3 | - | 2.3 | - | - | - | - | - | - | 0.10 | 5 |
| *Clausocalanus* sp. | C | - | - | - | - | - | - | - | 15.1 | 20.2 | 15.7 | - | 5.1 | - | - | - | - | - | - | - | - | 0.09 | 5 |
| *Centropagus* sp. | A | 5.2 | - | 12.1 | - | - | 1.5 | 3.3 | 14.3 | 7.1 | 0.5 | 8.4 | 1.1 | - | 1.4 | 1.6 | - | 1.7 | 1.1 | 1.4 | 3.8 | 0.02 | 8 |
| *Centropagus* sp. | B | 12.2 | 8.8 | 20.6 | - | - | 1.8 | 8.1 | 15.6 | 11.3 | 5.8 | 14.1 | 1.8 | - | 3.8 | 3.5 | - | 5.1 | 3.2 | 5.5 | 8.7 | 0.01 | 8 |
| *Centropagus* sp. | C | 3.6 | - | 3.2 | - | - | - | 1.4 | 4.2 | - | - | 1.9 | 1.1 | - | 0.2 | - | - | 0.6 | - | 0.3 | 0.9 | 0.06 | 8 |
|  |  |  |  |  |  |  |  |  |  |  |  |  |  |  |  |  |  |  |  |  |  |  |  |

**Supplementary Table 4A.** The *R^2^* and *p*-values of the linear regressions of DFAA and dissolved free taurine over time from the crustacean zooplankton in the Gulf of Alaska (GoA). A, B indicate the replicates of each experiment. The red color indicates an *R^2^* > 0.600 and a *p*-value > 0.05. (-) means, it was not possible to calculate a release rate. Abbreviations: St, station; R, replicates; Asp, Aspartic acid; Glu, Glutamic acid; Asn, Asparagine; Ser, Serine; Gln, Glutamine; His, Histine; Gly, Glycine; Arg, Arginine; Thr, Threonine; Ala, Alanine; Tau, Taurine; GABA, gamma aminobutyric acid; Tyr, Tyrosine; Met, Methionine; Val, Valine; Trp, Tryptophan; Phe, Phenylalanine; Ile, Isoleucine; Leu, Leucine; Lys, Lysine

| **St.** | **R** |  | **Asp** | **Glu** | **Asn/Ser** | **Asn** | **Ser** | **Gln** | **His** | **Gly** | **Arg** | **Thr** | **Ala** | **Tau** | **GABA** | **Tyr** | **Met/Val** | **Trp** | **Phe** | **Ile** | **Leu** | **Lys** |
| --- | --- | --- | --- | --- | --- | --- | --- | --- | --- | --- | --- | --- | --- | --- | --- | --- | --- | --- | --- | --- | --- | --- |
| 5 | A | *R^2^* | 0.940 | 0.987 | - | 0.964 | 0.964 | 0.892 | 0.893 | 0.898 | 0.975 | 0.951 | 0.952 | 0.963 | 0.762 | 0.864 | 0.955 | - | 0.943 | 0.970 | 0.982 | 0.977 |
|  |  | *p* | 0.000 | 0.000 | - | 0.000 | 0.000 | 0.001 | 0.004 | 0.001 | 0.000 | 0.000 | 0.000 | 0.000 | 0.010 | 0.002 | 0.000 | - | 0.000 | 0.000 | 0.000 | 0.000 |
| 5 | B | *R^2^* | 0.969 | 0.977 | 0.886 | - | - | 0.970 | 0.794 | 0.933 | 0.922 | 0.988 | 0.972 | 0.909 | - | 0.938 | 0.820 | - | 0.962 | 0.821 | 0.864 | 0.923 |
|  |  | *p* | 0.000 | 0.000 | 0.000 | - | - | 0.000 | 0.003 | 0.000 | 0.000 | 0.000 | 0.000 | 0.000 | - | 0.000 | 0.002 | - | 0.000 | 0.002 | 0.001 | 0.000 |
| 8 | A | *R^2^* | 0.951 | 0.987 | - | 0.956 | 0.884 | 0.951 | 0.951 | 0.965 | 0.985 | 0.982 | 0.934 | 0.985 | - | 0.921 | 0.969 | - | 0.992 | 0.930 | 0.920 | 0.920 |
|  |  | *p* | 0.005 | 0.001 | - | 0.004 | 0.017 | 0.005 | 0.005 | 0.003 | 0.008 | 0.001 | 0.007 | 0.001 | - | 0.010 | 0.002 | - | 0.000 | 0.008 | 0.010 | 0.010 |
| 12 | A | *R^2^* | 0.964 | 0.961 | 0.954 | - | - | 0.967 | 0.840 | 0.920 | 0.960 | 0.960 | 0.957 | 0.968 | - | 0.850 | 0.955 | - | 0.910 | 0.942 | 0.936 | 0.958 |
|  |  | *p* | 0.003 | 0.003 | 0.004 | - | - | 0.003 | 0.029 | 0.010 | 0.003 | 0.003 | 0.004 | 0.002 | - | 0.026 | 0.004 | - | 0.012 | 0.006 | 0.007 | 0.004 |
| 17 | A | *R^2^* | 0.930 | 0.926 | 0.914 | - | - | - | 0.641 | 0.921 | 0.941 | 0.777 | 0.960 | 0.880 | - | 0.944 | 0.883 | - | - | 0.890 | 0.920 | 0.943 |
|  |  | *p* | 0.002 | 0.002 | 0.011 | - | - | - | 0.056 | 0.002 | 0.001 | 0.020 | 0.001 | 0.006 | - | 0.001 | 0.005 | - | - | 0.005 | 0.002 | 0.001 |
| 20 | A | *R^2^* | 0.966 | 0.974 | - | 0.971 | 0.976 | 0.965 | 0.965 | 0.946 | 0.898 | 0.965 | 0.976 | 0.998 | - | 0.971 | 0.979 | - | 0.999 | 0.990 | 0.982 | 0.950 |
|  |  | *p* | 0.003 | 0.002 | - | 0.002 | 0.002 | 0.003 | 0.003 | 0.005 | 0.014 | 0.003 | 0.002 | 0.000 | - | 0.002 | 0.001 | - | 0.000 | 0.000 | 0.001 | 0.005 |
| 29 | A | *R^2^* | 0.961 | 0.972 | - | 0.855 | 0.905 | 0.985 | 0.959 | 0.927 | - | - | 0.875 | 0.856 | - | - | 0.959 | - | - | - | - | - |
|  |  | *p* | 0.003 | 0.002 | - | 0.024 | 0.013 | 0.001 | 0.004 | 0.009 | - | - | 0.020 | 0.024 | - | - | 0.004 | - | - | - | - | - |

**Supplementary Table 4B.** The *R^2^* and *p*-values of the linear regressions of DFAA and dissolved free taurine over time from the mixed copepod communities in the North Atlantic (NA). The ratio of taurine to the total DFAA (mol/mol) released is also indicated. A, B indicate the replicates of each experiment. The red color indicates an *R^2^* > 0.600 and a *p*-value > 0.05. (-) means, it was not possible to calculate a release rate. Abbreviations: MCC, mixed copepod community; R, replicates; Asp, Aspartic acid; Glu, Glutamic acid; Asn, Asparagine; Ser, Serine; Gln, Glutamine; His, Histine; Gly, Glycine; Arg, Arginine; Thr, Threonine; Ala, Alanine; Tau, Taurine; GABA, gamma aminobutyric acid; Tyr, Tyrosine; Met, Methionine; Val, Valine; Trp, Tryptophan; Phe, Phenylalanine; Ile, Isoleucine; Leu, Leucine; Lys, Lysine.

|  | **R** |  | **Asp** | **Glu** | **Asn/Ser** | **Asn** | **Ser** | **Gln** | **His** | **Gly** | **Arg** | **Thr** | **Ala** | **Tau** | **GABA** | **Tyr** | **Met/Val** | **Trp** | **Phe** | **Ile** | **Leu** | **Lys** |
| --- | --- | --- | --- | --- | --- | --- | --- | --- | --- | --- | --- | --- | --- | --- | --- | --- | --- | --- | --- | --- | --- | --- |
| MCC | A | *R^2^* | 0.831 | 0.915 | 0.813 | - | - | - | 0.701 | 0.771 | 0.812 | 0.872 | - | 0.815 | - | 0.757 | - | - | 0.892 | 0.787 | 0.775 | - |
|  |  | *p* | 0.011 | 0.003 | 0.014 | - | - | - | 0.038 | 0.021 | 0.014 | 0.006 | - | 0.014 | - | 0.024 | - | - | 0.005 | 0.018 | 0.021 | - |
| MCC | B | *R^2^* | - | 0.832 | - | - | - | - | - | 0.758 | 0.833 | 0.966 | 0.623 | 0.852 | - | - | - | - | - | - | - | - |
|  |  | *p* | - | 0.011 | - | - | - | - | - | 0.024 | 0.011 | 0.000 | 0.062 | 0.009 | - | - | - | - | - | - | - | - |
| MCC | C | *R^2^* | - | - | - | - | - | - | 0.874 | 0.919 | - | 0.959 | - | 0.971 | - | 0.869 | 0.846 | - | 0.916 | 0.937 | 0.850 | - |
|  |  | *p* | - | - | - | - | - | - | 0.006 | 0.003 | - | 0.001 | - | 0.000 | - | 0.007 | 0.009 | - | 0.003 | 0.001 | 0.009 | - |

**Supplementary Table 4C.** The *R^2^* and *p*-values of the linear regressions of DFAA and dissolved free taurine over time from the single species incubations in the North Atlantic (NA). The ratio of taurine to the total DFAA (mol/mol) released is also indicated. A, B,C indicate the replicates of each experiment. The red color indicates an *R^2^* > 0.600 and a *p*-value > 0.05; the orange indicates an *R^2^* < 0.600 and a *p*-value < 0.05. (-) means, it was not possible to calculate a release rate. Abbreviations: Sp, species; R, replicates; Asp, Aspartic acid; Glu, Glutamic acid; Asn, Asparagine; Ser, Serine; Gln, Glutamine; His, Histine; Gly, Glycine; Arg, Arginine; Thr, Threonine; Ala, Alanine; Tau, Taurine; GABA, gamma aminobutyric acid; Tyr, Tyrosine; Met, Methionine; Val, Valine; Trp, Tryptophan; Phe, Phenylalanine; Ile, Isoleucine; Leu, Leucine; Lys, Lysine.

| **Sp.** | **R** |  | **Asp** | **Glu** | **Asn/Ser** | **Asn** | **Ser** | **Gln** | **His** | **Gly** | **Arg** | **Thr** | **Ala** | **Tau** | **GABA** | **Tyr** | **Met/Val** | **Trp** | **Phe** | **Ile** | **Leu** | **Lys** |
| --- | --- | --- | --- | --- | --- | --- | --- | --- | --- | --- | --- | --- | --- | --- | --- | --- | --- | --- | --- | --- | --- | --- |
| *Calanus* sp. | A | *R^2^* | - | - | - | - | - | - | - | 0.719 | 0.731 | - | 0.527 | 0.826 | 0.646 | 0.527 | - | - | - | - | - | - |
|  |  | *p* | - | - | - | - |  | - | - | 0.016 | 0.007 | - | 0.042 | 0.002 | 0.029 | 0.042 | - | - | - | - | - | - |
| *Calanus* sp. | B | *R^2^* | - | - | - | - | - | - | 0.624 | 0.701 | - | 0.717 | - | 0.773 | - | 0.533 | - | - | - | - | 0.807 | 0.813 |
|  |  | *p* | - | - | - | - | - | - | 0.020 | 0.019 | - | 0.016 | - | 0.009 | - | 0.040 | - | - | - | - | 0.006 | 0.005 |
| *Calanus* sp. | C | *R^2^* | - | - | 0.566 | - | - | - | 0.739 | 0.622 | 0.606 | - | - | 0.852 | - | - | - | - | 0.888 | 0.765 | 0.811 | 0.703 |
|  |  | *p* | - | - | 0.031 | - | - | - | 0.006 | 0.020 | 0.023 | - | - | 0.001 | - | - | - | - | 0.000 | 0.004 | 0.002 | 0.009 |
| *Acartia* sp. | A | *R^2^* | - | - | - | - | - | 0.711 | 0.866 | 0.717 | - | 0.929 | - | 0.878 | - | - | - | - | - | - | - | - |
|  |  | *p* | - | - | - | - | - | 0.035 | 0.007 | 0.033 | - | 0.000 | - | 0.002 | - | - | - | - | - | - | - | - |
| *Acartia* sp. | B | *R^2^* | - | - | - | - | - | - | - | 0.799 | - | 0.775 | 0.818 | 0.878 | - | - | - | - | - | - | - | - |
|  |  | *p* | - | - | - | - | - | - | - | 0.007 | - | 0.009 | 0.005 | 0.002 | - | - | - | - | - | - | - | - |
| *Acartia* sp. | C | *R^2^* | - | - | - | - | - | - | - | 0.625 | 0.872 | 0.742 | - | 0.859 | - | - | - | - | - | - | - | 0.715 |
|  |  | *p* | - | - | - | - | - | - | - | 0.034 | 0.002 | 0.013 | - | 0.003 | - | - | - | - | - | - | - | 0.017 |
| *Clausocalanus* sp. | A | *R^2^* | - | - | - | - | - | - | - | 0.913 | - | - | 0.642 | 0.617 | - | - | - | - | - | - | - | - |
|  |  | *p* | - | - | - | - | - | - | - | 0.003 | - | - | 0.055 | 0.036 | - | - | - | - | - | - | - | - |
| *Clausocalanus* sp. | B | *R^2^* | - | - | - | - | - | - | - | 0.754 | 0.758 | - | 0.748 | 0.817 | - | 0.639 | - | - | - | - | - | - |
|  |  | *p* | - | - | - | - | - | - | - | 0.011 | 0.011 | - | 0.012 | 0.005 | - | 0.031 | - | - | - | - | - | - |
| *Clausocalanus* sp. | C | *R^2^* | - | - | - | - | - | - | - | 0.906 | 0.941 | 0.906 | - | 0.741 | - | - | - | - | - | - | - | - |
|  |  | *p* | - | - | - | - | - | - | - | 0.001 | 0.000 | 0.001 | - | 0.013 | - | - | - | - | - | - | - | - |
| *Centropagus* sp. | A | *R^2^* | 0.685 | - | 0.919 | - | - | 0.600 | 0.866 | 0.951 | 0.964 | 0.584 | 0.944 | 0.869 | - | 0.808 | 0.944 | - | 0.713 | 0.952 | 0.907 | 0.856 |
|  |  | *p* | 0.011 | - | 0.000 | - | - | 0.041 | 0.001 | 0.000 | 0.000 | 0.027 | 0.000 | 0.001 | - | 0.002 | 0.000 | - | 0.008 | 0.000 | 0.000 | 0.001 |
| *Centropagus* sp. | B | *R^2^* | 0.850 | 0.690 | 0.850 | - | - | 0.838 | 0.900 | 0.794 | 0.792 | 0.685 | 0.891 | 0.842 | - | 0.821 | 0.668 | - | 0.889 | 0.795 | 0.779 | 0.776 |
|  |  | *p* | 0.001 | 0.011 | 0.001 | - | - | 0.001 | 0.000 | 0.019 | 0.003 | 0.011 | 0.000 | 0.001 | - | 0.005 | 0.013 | - | 0.000 | 0.003 | 0.004 | 0.004 |
| *Centropagus* sp. | C | *R^2^* | 0.855 | - | 0.685 | - | - | - | 0.606 | 0.641 | - | - | 0.794 | 0.915 | - | 0.651 | - | - | 0.706 | - | 0.757 | 0.829 |
|  |  | *p* | 0.003 | - | 0.022 | - | - | - | 0.039 | 0.030 | - | - | 0.007 | 0.000 | - | 0.028 | - | - | 0.018 | - | 0.024 | 0.012 |

**Supplementary Table 5A.**  Average length and diameter in mm (± standard deviations) of the dominant mesozooplankton species of the Gulf of Alaska (GoA) and the North Atlantic (NA) used in the community-specific incubation experiments.

| **Station** | **Cruise** | **Replicate** | ***N. cristatus*** | | ***Themisto* sp.** | | ***Vibilia* sp.** | |
| --- | --- | --- | --- | --- | --- | --- | --- | --- |
|  |  |  | **Length** | **Diameter** | **Length** | **Diameter** | **Length** | **Diameter** |
| 5 | GoA | A | 4.5±1.5 | 1.3±0.4 | 5.6±1.2 | 1.5±0.3 | 6.7±0.5 | 1.5±0.3 |
|  |  | B | 4.8±1.4 | 1.5±0.4 | 5.6±1.0 | 1.6±0.2 | 6.9±1.2 | 1.7±0.3 |
| 8 | GoA |  | - | - | 4.2±0.8 | 1.2±0.2 | 8.0±1.9 | 1.8±0.3 |
| 12 | GoA |  | 6.3±0.4 | 2.0±0.3 | 4.5±1.5 | 1.3±0.4 | 10±1.8 | 2.2±0.4 |
| 17 | GoA |  | 6.3±0.9 | 1.9±0.2 | 4.2±0.6 | 1.2±0.3 | 5.5 | 1.5 |
| 20 | GoA |  | 6.5±0.4 | 1.9±0.2 | 5.0±1.7 | 1.4±0.6 | 3.8±1.5 | 1.2±0.1 |
| 29 | GoA |  | 3.7±1.4 | 1.5±1.7 | 4.9±1.0 | 1.4±0.4 | 7.7±0.7 | 2.1±0.2 |
|  |  |  | ***Acartia* sp.** | | ***Calanus* sp.** | | ***Centropagus* sp.** | |
| 14 | NA | A | 0.7±0.2 | 0.2±0.1 | 0.6±0.1 | 0.2±0.1 | 0.7±0.2 | 0.3±0.1 |
|  |  | B | 0.8±0.2 | 0.3±0.1 | 0.6±0.2 | 0.2±0.1 | 0.7±0.2 | 0.3±0.1 |
|  |  | C | 0.6±0.1 | 0.2±0.0 | 0.5±0.1 | 0.2±0.1 | 0.6±0.2 | 0.2±0.1 |

**Supplementary Table 5B.** Average length and diameter in mm (± standard deviation) of the copepod species in the North Atlantic (NA) used in the single species incubations to determine the species-specific Tau release rates; A, B, C indicate the replicates of each experiment; number of replicate measurements ranges between 30 - 37 individuals per species.

| **Station** | **Species** | **Replicate** | **Length** | **Diameter** |
| --- | --- | --- | --- | --- |
| 111 | *Calanus* sp. | A | 0.7±0.2 | 0.3±0.0 |
|  |  | B | 0.7±0.2 | 0.3±0.1 |
|  |  | C | 0.7±0.2 | 0.3±0.1 |
| 59 | *Acartia* sp. | A | 0.8±0.1 | 0.3±0.1 |
|  |  | B | 0.8±0.2 | 0.2±0.1 |
|  |  | C | 0.8±0.1 | 0.3±0.1 |
| 115 | *Clausocalanus* sp. | A | 0.6±0.2 | 0.3±0.1 |
|  |  | B | 0.7±0.2 | 0.2±0.1 |
|  |  | C | 0.6±0.1 | 0.2±0.1 |
| 111 | *Centropagus* sp. | A | 0.8±0.2 | 0.3±0.1 |
|  |  | B | 0.8±0.2 | 0.3±0.1 |
|  |  | C | 0.8±0.2 | 0.3±0.1 |

**Supplementary Table 6.** Copepod abundance data obtained from literature to estimate bulk taurine release rates and turnover time for the copepod community in the Pacific and Atlantic.

| **Ocean** | **Depth [m]** | **Copepod abundance [individuals m^-3^]** | | **Reference** |
| --- | --- | --- | --- | --- |
|  |  |  | |  |
| West subarctic Pacific | 0 - 100 | 3509 - 5082 | | Yamaguchi et al. (2002) |
|  |  | |  |  |
| Northeast Atlantic | 0 - ~100 | 1945 - 4451 | | Fernández de Puelles et al. (1996) |
|  |  |  | |  |

**Supplementary Table 7**. The effect size (Hedges *g*) between the different experiments of the Gulf of Alaska (GoA; *n* = 7) and the North Atlantic (NA; *n* = 3 for each experiment). The range of *g* is 0 - 1 and indicate the effect size between the experiments: *g* < 0.2 indicates small effect*, g* > 0.5 medium effect, and *g* > 0.8 strong effect.

| **In µmol g^-1^ C-biomass h^-1^** | Mixed copepod community | *Calanus* sp*.* | *Clausocalanus* sp*.* | *Acartia* sp*.* | *Centropagus* sp*.* |
| --- | --- | --- | --- | --- | --- |
| Mixed copepod community | - | 0.62 | 0.85 | 0.87 | 0.93 |
| *Calanus* sp*.* | - | - | 0.63 | 0.53 | 0.89 |
| *Clausocalanus* sp*.* | - | - | - | 0.66 | 0.89 |
| *Acartia* sp*.* | - | - | - | - | 0.90 |
| Mixed zooplankton community GoA | 0.97 | 0.95 | 0.94 | 0.94 | 0.53 |
| **In pmol individual^-1^ h^-1^** |  |  |  |  |  |
| Mixed copepod community | - | 0.77 | 1.00 | 1.00 | 1.00 |
| *Calanus* sp*.* | - | - | 0.97 | 0.99 | 0.99 |
| *Clausocalanus* sp*.* | - | - | - | 0.71 | 0.69 |
| *Acartia* sp*.* | - | - | - | - | 0.97 |
| Mixed zooplankton community GoA | 0.93 | 0.93 | 0.93 | 0.93 | 0.93 |
